# Mechanical Alloying as a Way to Produce Metastable Single-Phase High-Entropy Alloys beyond the Stability Criteria

**DOI:** 10.3390/nano14010027

**Published:** 2023-12-21

**Authors:** Lucía Santiago-Andrades, Antonio Vidal-Crespo, Javier S. Blázquez, Jhon J. Ipus, Clara F. Conde

**Affiliations:** Departamento de Física de la Materia Condensada, ICMSE-CSIC, Universidad de Sevilla, P.O. Box 1065, 41080 Sevilla, Spain

**Keywords:** high-entropy alloys, mechanical alloying, solid solution, metastability

## Abstract

Various stability criteria developed for high-entropy alloys are applied to compositions produced by mechanical alloying. While they agree with the annealed samples, these criteria fail to describe the as-milled metastable systems, highlighting the ability of mechanical alloying to overcome the limitations imposed by these criteria. The criteria are based on atomic size (Ω ≥ 1.1 and $\delta_{r}\;\leq \;6$.6%) and/or electronegativity misfit, as well as on mixing enthalpy ($\Lambda >0.95\;J\;{mol}^{-1}K^{-1}$ and $-5\;kJ\;{mol}^{-1}<∆H_{mix}<0$), or purely thermodynamic ($\phi_{Ye}>20$; $\phi_{King}>1$; $T_{eff}<500\;K$). These criteria are applied to several compositions found in the literature and to two metastable fcc solid solutions produced by mechanical alloying with compositions Al_0.75_CoXFeNi with X = Cr and Mn. Single-phase microstructures are stable up to above 600 K, leading to more stable multiphase systems after annealing above this temperature. Mössbauer spectrometry shows that, whereas the alloy with Cr is paramagnetic in the as-milled and annealed state, the alloy with Mn changes from paramagnetic to ferromagnetic behavior (Curie temperature ~700 K) after annealing. Thermomagnetic experiments on annealed samples show for both compositions some hysteretic events at high temperatures (850 to 1000 K), probably ascribed to reversible ordering phenomena.

## 1. Introduction

Almost 20 years ago, Cantor [1] and Yeh [2] opened a new branch in Materials Science with the development of high-entropy alloys (HEA) [3]. HEAs are multielement systems that derive their name from the high configurational entropy directly estimated from the composition $∆S_{conf}=-R\sum x_{i}lnx_{i}$, where $x_{i}$ represents the atomic concentration of the i-th element and R is the gas constant. For an equiatomic quinary alloy, $∆S_{conf}=1.6R=13.4\;JK^{-1}{mol}^{-1}$, and a compromise threshold of $∆S_{conf}>1.5R$ is considered for defining HEAs, allowing for the inclusion of quinary alloys slightly deviating from equiatomic compositions [4]. This threshold can be further relaxed to encompass quinary alloys with $0.05<x_{i}<0.35$ with $∆S_{conf}\geq 1.36R$ [5], thus also incorporating quaternary equimolar alloys. However, it is important to note that the $∆S_{conf}$ value is valid and physically meaningful only for fully-disordered single-phase solid solutions, which is not always the case. A more stringent definition requires the formation of a single-phase solid solution with bcc, fcc, or hcp unit cells and a monoatomic motif. To provide clarity, Miracle and Senkov [5] confined HEAs to single-phase solid solutions and coined other terms, such as Cantor alloys, multi-principal element alloys, complex concentrated alloys, and baseless alloys, to categorize alloys within the extensive compositional space created by multielement systems, regardless of their multiphase nature.

In HEAs, the high value of $∆S_{conf}$ plays a crucial role in stabilizing the solid solution in contrast to the segregation of pure elements or the formation of intermetallic compounds, both of which offer significantly lower configurational entropy than a solid solution. The segregation of elements is favored when the individual elements exhibit positive enthalpies of mixing (estimated from binary systems as $∆H_{mix}=\sum_{i\neq j}4∆H_{ij}x_{i}x_{j}$, with $∆H_{ij}$ denoting the mixing enthalpy of a binary melt alloy with elements i and j [6]). On the other hand, the formation of intermetallic compounds occurs when $∆H_{mix}<<0$. Therefore, $∆H_{mix}\sim 0$ is the ideal situation to obtain an HEA. Consequently, King et al. [7] proposed a straightforward thermodynamic criterion for the stabilization of HEA, defining $\phi_{King}=-∆G_{SS}/\left|∆G_{max}\right|$, i.e., the ratio between Gibbs free energies of the solid solution, $∆G_{SS}$, and the most extreme absolute value among the potential binary systems in the composition (either $∆G_{max}>0$, leading to segregation, or $∆G_{max}<0$, leading to intermetallic formation). Therefore $\phi_{King}\geq 1$ was suggested for stable HEAs.

Regarding the stability of single-phase solid solution HEAs, various parameters, in addition to $∆S_{conf}$ and $∆H_{mix}$ [8,9,10], have been taken into consideration. Martin et al. [11] gathered these diverse parameters and developed the HEAPS software tool for predicting the stability and microstructure of multielement systems. The High-Entropy Alloys Predicting Software (HEAPS) project, is led by Pablo Martin St Laurence, from the Politechnical University of Catalonia (Spain), in collaboration with members of the Technical University Federico Santa María (Chile). Some of these parameters can be derived from single atom properties, such as atomic size misfit:

(1) $$\delta_{r}=\sqrt{\sum_{i=1}^{n}x_{i}{\left(1-\frac{r_{i}}{r}\right)}^{2}},$$

where $r_{i}$ and $x_{i}$ are the atomic radius and fraction of element i, and r is the average radius. Furthermore, other parameters, like $∆H_{mix}$ and $\left|∆G_{max}\right|$, are determined based on the potential binary alloys formed by the elements within the composition. 

Table 1 collects the parameters currently used to establish thresholds to predict the solid solution stability. Recently, some of the present study’s authors proposed a simple phenomenological model based on the averaging of the coefficients of the metallic bonding potential [12]. One advantage of this simple model lies in its reliance on single element properties, in contrast to the broader description derived from the properties of binary alloys. The metallic bonding can be described by a potential energy in the following form:

(2) $$U\left(r\right)=-\frac{A}{d}+\frac{B}{d^{2}},$$

where d is the distance to nearest neighbors, and A and B can be phenomenologically determined for a specific alloy using the equilibrium distance $d=d_{0}$ and the bulk modulus. The theoretical basis of Equation (2) is rooted in the stabilization of single metal atoms [13], where the attractive term accounts for the attraction of the core ion to the valence electrons shared to the Fermi gas; and the repulsive term arises from the confinement energy of that Fermi gas.

By employing average coefficients, an estimation of the equilibrium potential difference with respect to segregation, denoted as $∆U_{0}$, can be parametrized as an effective temperature, $T_{eff}=∆U_{0}/∆S_{conf}$. When comparing this model across different multicomponent compositions, a threshold of $T_{eff}\sim 500\;K$ emerges. Below this threshold, single-phase fcc phases were observed, whereas for $T_{eff}>500\;K$, bcc/B2 or multiphase systems were identified for a set of 70 AlCoCrCuFeNi alloys [12]. 

This analysis was extended to other compositions, encompassing quinary and sexinary alloys, involving a total of 16 elements, as referred to by Gorban et al. [15]. It is worth noting that in the case of the equiatomic ReMoWNbTa alloy, a single alloy with a bcc phase was observed with $T_{eff}<500\;K$, but with a low atomic radii misfit ($\delta_{r}\sim 2\%$). It is important to mention that distinguishing disordered bcc from ordered (or partially-ordered) B2 phases can be challenging due to similar average atomic scattering factors. However, the ordered structure may significantly affect the required parameter $\delta_{r}$ due to the accommodation of larger and smaller atoms on different sites. Nevertheless, this site preference causes the entropy value to deviate from the estimated $∆S_{conf}$, assuming the global composition.

**Figure 1:**
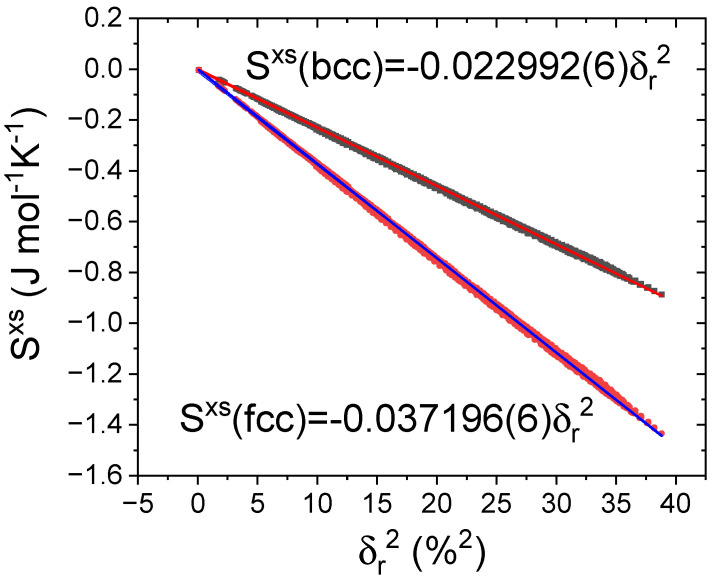
Excess entropy in bcc (dark yellow, data; red line, linear fitting) and fcc (pink, data; blue line, linear fitting) solid solutions as a function of the square of the atom size misfit for the CoFeNiCr_y_Mn_z_Al_x_ family within the ranges 0<x<1, 0<y<1, and 0<z<1 (1331 compositions obtained from HEAPS software [11]). Lines correspond to linear fitting of the data with regression coefficient $R^{2}=$ 0.99990.

In fact, even in single-phase solid solution systems, the assumption of complete disorder used for $∆S_{conf}$ is an idealization. $∆S_{conf}$ corresponds to an overestimated limit because the larger atoms tend to distort the lattice in such a way that they are preferentially surrounded by smaller ones. The excess entropy, $S^{xs}$, was estimated within the framework of the hard sphere model by Mansoori et al. [16]. Despite its complex dependence on $x_{i}$ and $r_{i}$ of the constituents, as well as on the packing fraction, f, (see [11,16]), simple linear relations with ${\delta_{r}}^{2}$ can be approximated for bcc and fcc structures (though the slope slightly varies for different alloy series). Figure 1 shows this linear fitting (regression coefficient >0.9999) for the CoFeNiCr_y_Mn_z_Al_x_ family within the ranges 0<x<1, 0<y<1, and 0<z<1.

Despite the many efforts made, as mentioned earlier, to elucidate the stability of solid solutions in multicomponent alloys, it is well known that supersaturated solid solutions can be traditionally obtained either by rapid quenching [17,18] or by mechanical alloying [19]. Nowadays, new techniques are used to produce metastable systems such as additive manufacturing [20] and laser melting [21]. Concerning mechanical alloying, this technique significantly expands the solubility limits in alloys and thus plays a role in enabling the formation of high-entropy alloys (HEAs) beyond what is predicted for thermodynamically-stable conditions. The development of metastable systems is justified from an application perspective due to the expected enhancement in mechanical properties in HEA systems. This potential was recognized by the group of Murty et al. [22], who pioneered the production of HEAs by mechanical alloying in 2008 [23].

While the number of articles on the mechanical alloying production of HEAs rapidly increased from 10 papers over a five-year period from 2008 to 2012 to nearly 100 in 2022, this surge has led to the publication of several reviews with a focus on HEA produced by mechanical alloying. Notable among them are the works from Vaidya et al. [22], Koch [24], and Torralba et al. [25]. It is also worth mentioning the section Suryanarayana dedicated to HEA systems in his recent review on mechanical alloying [26] and a recent review from the pioneering group on mechanically alloyed HEAs [27]. Suryanarayana discussed the validity of thermodynamical criteria adopted for rapid quenching and their applicability to mechanical alloyed systems. Concerning HEAs, he proposed a new criterion using the Darken–Gurry plots, where electronegativity difference is plotted versus atomic size difference. These plots predict the formation of solid solutions for specific $\delta_{r}$ values, with fcc structure occurring when there is a low difference in electronegativity, and bcc structure forming in cases of higher electronegativity differences. This representation is suggested to be valid not only for HEAs produced by mechanical alloying but also for those produced using other techniques. 

Unlike production techniques starting from the molten alloy, the mechanical alloying route can produce a supersaturated solution at low temperatures. Therefore, the stability criteria based on high-temperature thermodynamically-stable systems are not a priori adequate. Certainly, rapid quenching may also produce supersaturated solutions (metastable solid solutions) as a frozen microstructure from those thermodynamically stable at high temperature. In these cases, stability criteria can be adapted considering the entropic contribution to the free energy balance at the melting point (and, in fact, they are generally used in this way), which maximizes the effect of configurational entropy on the Gibbs energy.

In this work, we employed mechanical alloying of pure powders to obtain two distinct metastable quinary HEA compositions: Al_0.75_CoCrFeNi and Al_0.75_CoMnFeNi (in the following: Cr-alloy and Mn-alloy, respectively). It is worth noting that the former composition is known to present a biphasic character when produced by arc melting, resulting in a mixture of bcc and fcc phases [28]. The results are compared to recent data from the literature to highlight the disparities that arise from mechanical alloying in contrast to the predictions made according to the stability criteria currently in use.

## 2. Materials and Methods

Masses of 5 g of pure powders (>99.5%) were mixed to form compositions Al_0.75_CoCrFeNi and Al_0.75_CoMnFeNi (in the following: Cr- and Mn-alloy, respectively) and incorporated in 80 cm^3^ hardened steel bowls with 60 g of 10 mm steel balls. The milling process was conducted using a Fritsch Pulverisette P4 Vario planetary mill (Fritsch, Idar-Oberstein, Germany) at 250 rpm disc frequency and 500 rpm bowl frequency, with milling performed in 30-min intervals followed by 15-min pause steps. Sample preparation and extraction were carried out in argon atmosphere in a Saffron Ω glove box (Saffron, London, UK) to control oxygen and humidity levels. The times for extracting samples were chosen according to our previous experiments under these milling conditions. Initially, the powder size decreases from 1 h to 10 h of milling, but then cold welding is the predominant effect and powder size increases up to 1 mm after 30 h of milling. Due to the ductility of the systems, after 50 h of milling, the powder is almost completely adhered to the milling media, making it impossible to extract any samples for analysis. 

The composition of the powders was analyzed by means of X-ray fluorescence, XRF, in an Eagle III microfluorescence device (EDAX, Mahwah, NJ, USA) with Rh anticatode. The microstructure was studied via X-ray diffraction, XRD, using Cu Kα radiation in a Bruker D8 Advance A25 powder diffractometer (Bruker, Karlsruhe, Germany), and Mössbauer spectrometry, using a Wissel spectrometer (Wissel, Starnberg, Germany) in transmission geometry with a 57Co source. NORMOS software (MS-DOS version) was used to fit the spectra (both SITE and DIST programs) [29]. 

The thermal stability was studied by means of differential scanning calorimetry, DSC, in a Perkin-Elmer DSC7 calorimeter (Perkin-Elmer, Norwalk, CT, USA) calibrated using Pb and K_2_CrO_4_ standards and by thermomagnetic gravimetry, TMG, in a Perkin-Elmer TGA7 (Perkin-Elmer, USA), calibrated using the Curie temperatures of alumel (436 K), Ni (627 K), and Fe (1053 K) standards. Magnetic properties were assessed using Mössbauer spectrometry, TMG, and a vibrating sample magnetometer, VSM, in a LakeShore 7407 (LakeShore, Carson, CA, USA), calibrated with a standard sample of Ni. Samples for DSC and magnetic measurements were pressed in a uniaxial Atlas Manual Hydraulic Press (Specac, Orpington, UK) applying 2 tons on discs of 5 mm diameter. This prevented the movement of the powder due to magnetic field. 

The HEAPS software tool developed by Martin et al. [11] was used to calculate the different stability parameters, except when explicitly indicated otherwise. 

## 3. Results

The experimental compositions determined through XRF closely align with the nominal compositions. The Fe and Cr contents may slightly increase due to contamination from the milling media (in the Cr-free composition, the Cr content remains below 0.5% after 30 h milling). Both Co and Ni exhibit a similar trend, with a slight decrease to compensate for the increase in Fe and Cr content. Finally, Al seems to progressively decrease with milling time, although, as the lightest element among those studied here, its measurement carries a larger margin of error. The estimated experimental compositions after 30 h of milling are Al_0.7_Co_1.0_Cr_1.0_Fe_1.0_Ni_0.9_ and Al_0.76_Co_0.96_Cr_0.02_Mn_1.00_Fe_1.06_Ni_0.96_ for the Cr- and Mn-alloys, respectively. 

**Figure 2:**
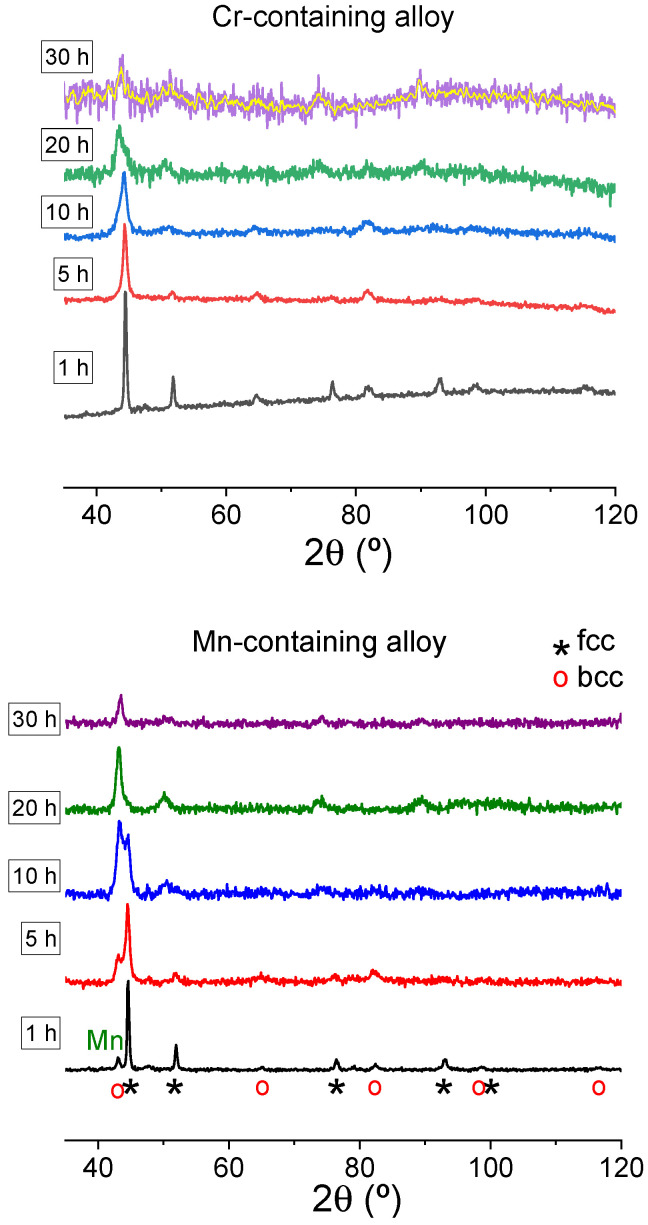
XRD patterns of Al_0.75_CoXFeNi alloys (X = Cr, upper panel, and Mn, lower panel) as a function of milling time. The noisy XRD pattern of the 30 h milled Cr-alloy sample is due to the large powder particle size, which impedes a good flat surface.

Figure 2 displays the XRD patterns of the as-milled samples after different times of milling for both compositions. In the early stages of milling, the pure phases disappear, and atoms quickly integrate in fcc or bcc phases. However, in the case of Mn-alloy, some Mn-rich phase remains. As milling progresses, the bcc phase fraction reduces, and after 20 h of milling, only a single fcc phase is detected. The lattice parameters of the fcc phase are similar for both compositions (a= 3.62 ± 0.01 Å after 20 h of milling).

The lower limits for crystal size of the fcc phase were determined from the broadening of the (111) maximum (decoupled from the (110) maximum of the bcc phase using two pseudoVoigt functions when necessary). Notably, while the crystal size of the Cr-alloy progressively decreases as milling progresses (from 68 ± 7 to 18 ± 3 nm as milling time increases from 10 to 30 h), this parameter remains stable for the Mn-alloy (about 90–100 nm). 

**Figure 3:**
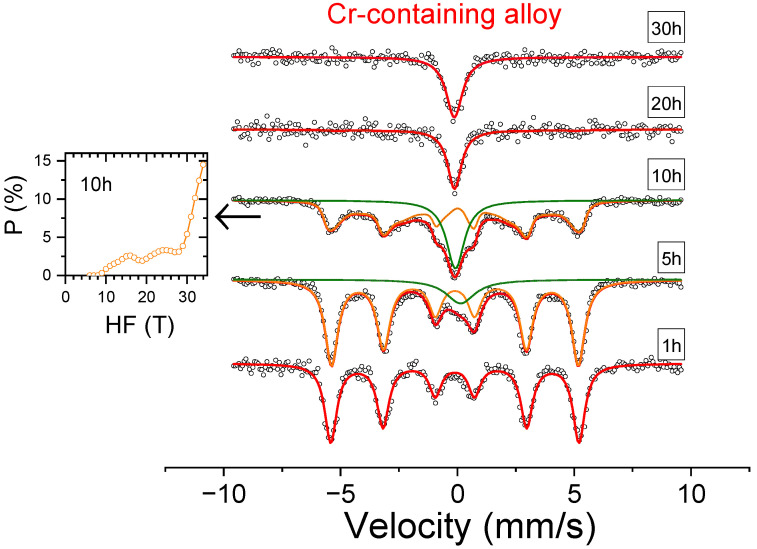
Mössbauer spectra of Al_0.75_CoCrFeNi alloy as a function of milling time. Circles correspond to experimental data. Thick red lines correspond to total fitting. Orange curves correspond to ferromagnetic contribution, and green curves correspond to paramagnetic contribution. Inset shows the probability of ferromagnetic contribution for a sample milled for 10 h, the spectrum of which was fitted using a distribution. Other contributions were fitted using a single site.

**Figure 4:**
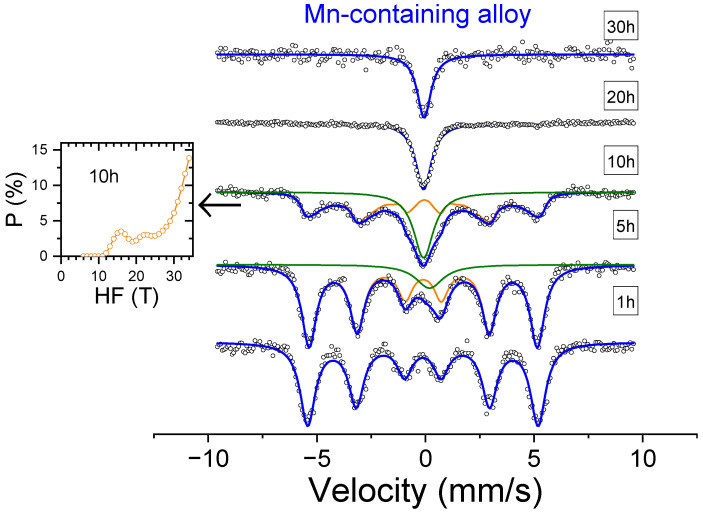
Mössbauer spectra of Al_0.75_CoMnFeNi alloy as a function of milling time. Circles correspond to experimental data. Thick blue lines correspond to total fitting. Orange curves correspond to ferromagnetic contribution, and green curves correspond to paramagnetic contribution. Inset shows the probability of ferromagnetic contribution for a sample milled for 10 h, the spectrum of which was fitted using a distribution. Other contributions were fitted using a single site.

The Mössbauer spectra of the as-milled samples are shown in Figure 3 and Figure 4 as a function of milling time. In agreement with the XRD results, the ferromagnetic contribution, ascribed to an FeCo-based bcc phase, gradually diminishes with ongoing milling, while the paramagnetic contribution, linked to the integration of Fe in the fcc phase, increases. After 20 h of milling, the contribution to the spectra of Fe atoms in ferromagnetic sites is negligible for both compositions.

**Figure 5:**
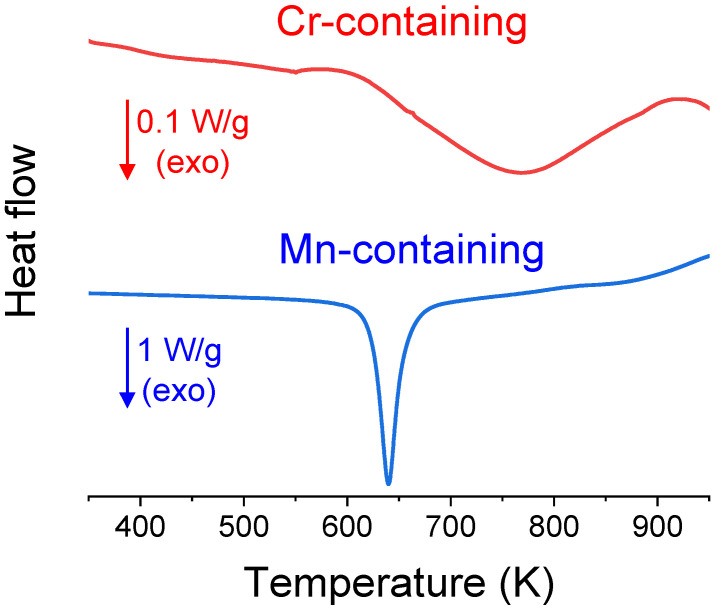
DSC scans at 20 K/min of Al_0.75_CoXFeNi alloys (X = Cr, upper curve, and Mn, lower curve) for as-milled samples after 30 h of milling. Note that the upper curve is zoomed ×10 with respect to lower curve.

The stability of the single-phase fcc solid solution achieved after 30 h of milling was studied by means of DSC scans, as depicted in Figure 5. The processes shown are irreversible, as a second run does not reveal any additional deviations from baseline. This figure shows that the Mn-alloy displays a more well-defined transformation with a peak temperature at 640 K and an enthalpy of transformation of 162 J/g. In contrast, the Cr-alloy exhibits a much broader transformation, with a peak temperature at 768 K and a transformation enthalpy of 54 J/g.

**Figure 6:**
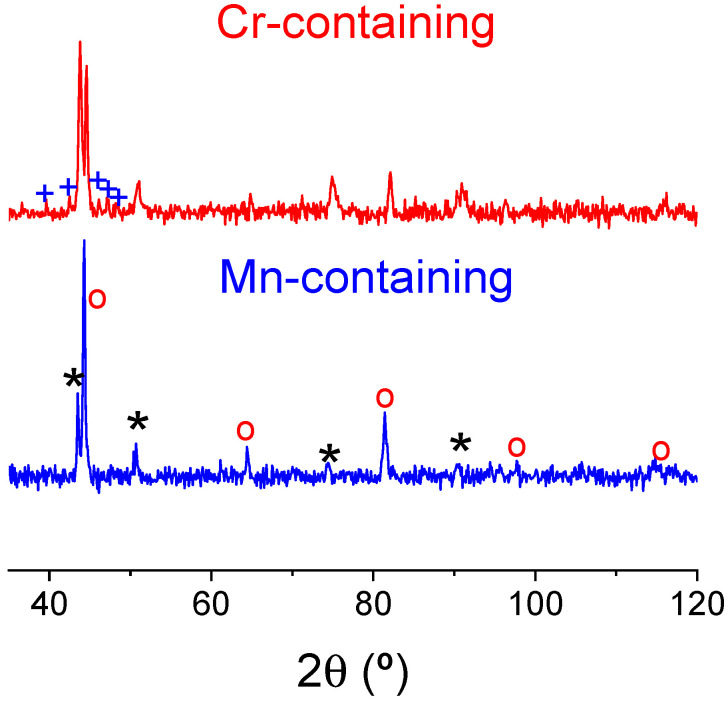
Room-temperature XRD patterns after DSC experiments. Black asterisks correspond to fcc phase; red circles correspond to bcc phase; and blue crosses in Cr-alloy identify the σ phase.

In order to understand the effect of these transformations, XRD experiments (Figure 6) were performed at room temperature after DSC scans (maximum heating temperature, 973 K). The XRD patterns confirm the irreversible nature of the transformations detected via DSC. The microstructures of the annealed samples exhibit a multiphase character, with the coexistence of bcc and fcc phases. Additionally, a sigma phase is detected for the Cr-alloy. This finding aligns with results reported for annealed AlCoCrFeNi at 1073 K after short-duration milling [30]. In qualitative agreement with the transformation enthalpy measured via DSC, the transformed fraction from fcc to bcc phase is higher in the case of the Mn-alloy.

**Figure 7:**
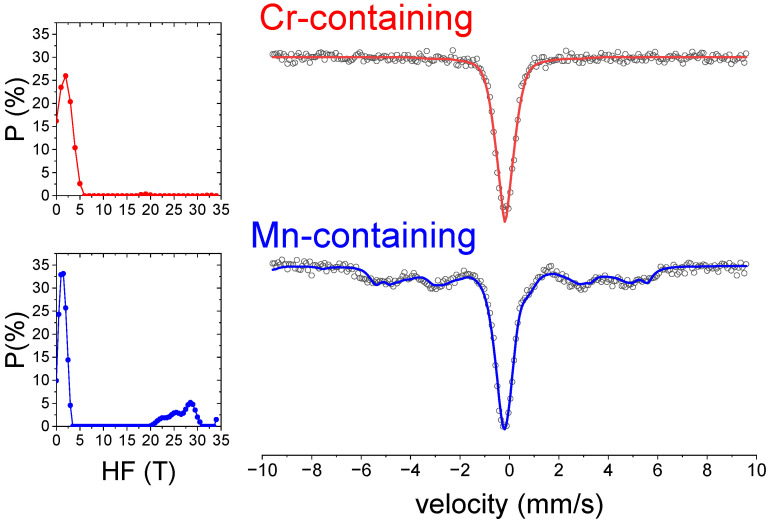
Right panel: Mössbauer spectra of 30 h milled samples after heating to 973 K during DSC. Symbols correspond to experimental data, and lines correspond to fitting using a distribution of hyperfine fields (shown in the corresponding left panels).

The Mössbauer spectra of annealed samples (Figure 7), after heating to 973 K during DSC, indicate that Fe atoms mainly remain in the fcc paramagnetic phase. In the case of the Cr-alloy, no ferromagnetic Fe sites are detected, while for the Mn-alloy, some magnetically-ordered contribution is observed, which corresponds with the larger amount of bcc phase in this composition.

**Figure 8:**
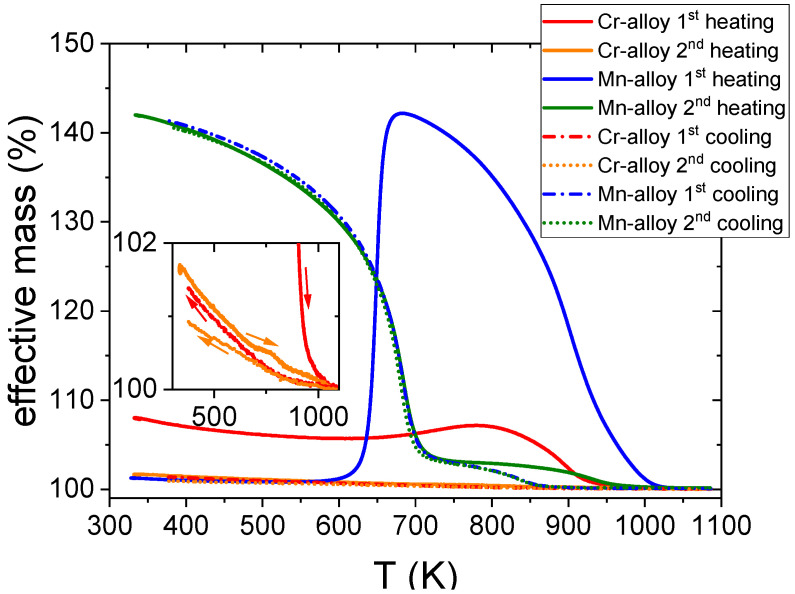
TMG scans at 33 K/min of 30 h milled samples. Inset shows a zoomed *Y*-axis to appreciate the behavior of the Cr-alloy sample. Arrows indicate the sense of temperature change.

Figure 8 shows TMG heating scans at 33 K/min for 30 h as-milled samples, followed by cooling to room temperature and a subsequent second cycle. The temperatures reached were above the limit temperature of DSC. 

In the case of the Mn-alloy, the as-milled sample exhibits weak ferromagnetic behavior (probably due to impurities). Coinciding with the DSC event associated with the development of the bcc phase, a substantial increase in magnetization occurs. Following this rise, detected during the first heating, the magnetization decreases to zero at ~1000 K for the Mn-alloy. However, this decline is not solely attributed to approaching the Curie temperature but may also result from structural transformations (e.g., ordering). In fact, the cooling process registered a first increase in magnetization at ~850 K and a clear Curie transition at ~700 K. In the second cycle, this reversible process occurs at 700 K, but on heating, a decrease in magnetization is observed at 950 K. During cooling, this phenomenon reappears at 700 K (as observed in the first cooling cycle), indicating that the high-temperature decrease in magnetization corresponds to a hysteretic process. Recently, Gao et al. [31] showed that the presence of Al induces a transition from antiferromagnetic to ferromagnetic coupling in Mn atoms, resulting in soft magnetic behavior in Co_4_Fe_2_Mn_1.5_Al_1.5_ alloy, which exhibits a B2 ordered structure. This could also be occurring once the as-milled sample of Mn-alloy is annealed, leading to thermal stabilization of the Al positions.

Regarding the Cr-alloy, although the magnetization is higher for the as-milled samples, there is no sudden process during the first heating, which aligns with the DSC scan. At around 925 K, the magnetization drops to zero in a similar way to that observed in the Mn-alloy. With further cooling, a rise in magnetization is not detected at this temperature but, rather, a gradual increase starting at approximately 800 K. Subsequent heating shows a tiny effect at ~750 K, which is not observed during the first cooling or the second cooling. However, this effect is reversible, as it is observed in subsequent heating scans (not shown here). Therefore, similar to that in the Mn-alloy, it appears that some hysteretic process occurs at elevated temperatures in the Cr-alloy.

**Figure 9:**
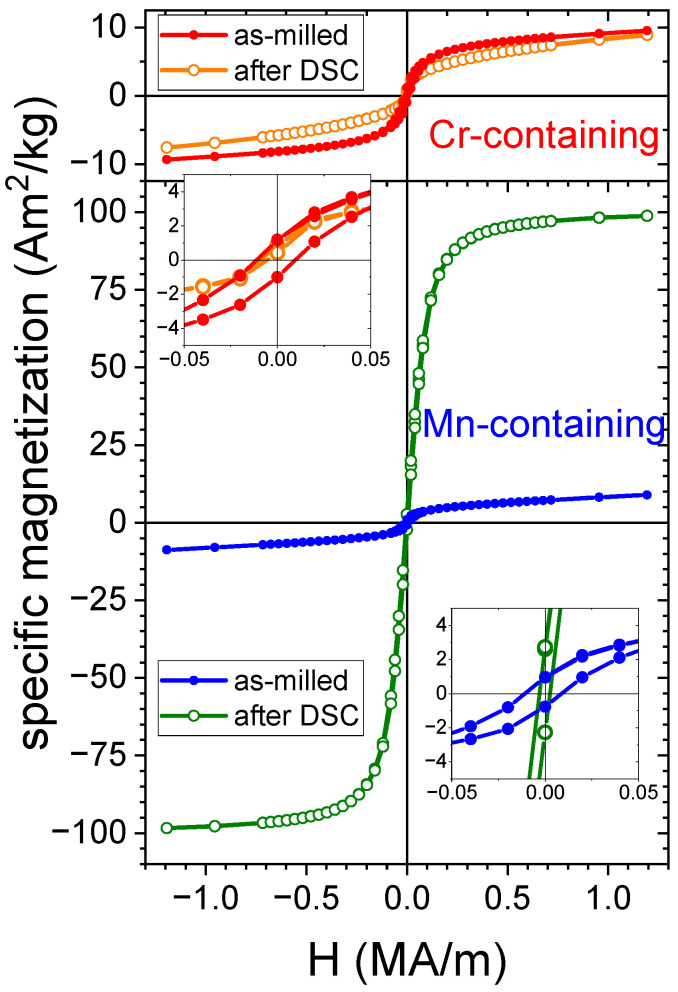
Room-temperature hysteresis loops from VSM for 30 h as-milled samples and after heating to 973 K in DSC.

Room-temperature magnetic hysteresis loops were obtained by means of VSM for 30 h milled samples in both as-milled and annealed states. Figure 9 shows that both the Cr-alloy samples and the as-milled Mn-alloy exhibit low specific magnetization (σ = 9 emu/g at 1.5 T), which aligns with the finding from the Mössbauer spectra. After annealing in the DSC, σ for the Mn-alloy increases by approximately one order of magnitude (up to ~100 emu/g at H= 1.194·10^6^ A/m; $\mu_{0}H=$ 1.5 T), in agreement with the clear ferromagnetic contribution detected by means of Mössbauer spectroscopy and with the TMG experiments discussed earlier. In contrast, the Cr-alloy experiences a slight decrease in magnetization after annealing, also in agreement with Mössbauer and TMG results.

Even in samples with low magnetization, there is a high susceptibility at low fields, which could be attributed to ferromagnetic impurities contributing to a change in specific magnetization of $∆\sigma_{imp}\sim$5 Am^2^/kg. This impurity phase was solely detected by means of TMG, where nonzero magnetization was observed at room temperature. By considering the saturation magnetization of pure Fe ($M_{S}=$ 1.71·10^6^ A/m) and its density (ρ= 7874 kg/m^3^), the $∆\sigma_{imp}$ value would imply ~2 at. % of Fe impurities. This can explain why such a small amount (within the error range of XRF) was not detected by means of either XRD or Mössbauer spectrometry.

For both samples, magnetic softening is observed after annealing in the DSC, although the coercive field is below the precision limit of our equipment. This property is very sensitive to both size scales in powder particles—powder particle size and crystal size—leading to two different magnetoelastic contributions to the magnetic anisotropy [32]. Concerning the soft magnetic applicability of the samples produced in this study, Mn-containing alloy milled for 30 h after annealing (bcc+fcc phases) exhibits a high saturation magnetization and a low coercivity.

## 4. Discussion

Previous experimental data on arc-melted alloys indicated the presence of multiphase systems, with coexisting bcc and fcc phases for the Cr-alloy composition [28]. However, in our experiments, we achieved single-phase supersaturated fcc solid solutions after milling. Subsequent annealing leads to the formation of a multiphase structure to stabilize the system. Various criteria have been proposed to predict the microstructure in multi-principal element alloys. In the following, some of these criteria are applied to the studied compositions. The metastability of the single-phase fcc solid solution and its further stabilization as multiphase bcc+fcc solid solutions by annealing, as well as the presence of a sigma phase in the Cr-alloy, are discussed within the framework of these criteria.

Table 2 compiles the values of different parameters proposed to determine the stability of the solid solution in the HEAs for the nominal compositions. No significant changes are observed after considering experimental compositions, making it valid to discuss in terms of the nominal compositions.

Martin et al. [11] classified the different criteria for single-phase solid solution stabilization into those that determine solid solution or intermetallic formation and those that predict the lattice structure of the solid solution. Regarding the former classification, the most widely used criterion is the one proposed by Yang et al. based on the representation of Ω vs. $\delta_{r}$ [10]: solid solution is predicted when Ω ≥ 1.1 and $\delta_{r}$ ≤ 6.6%. Table 2 shows that Ω = 2.1 and 1.7 and $\delta_{r}$ = 5.3 and 5.5 for the Cr- and Mn-alloys, respectively. These values position these compositions well within the expected solid solution region. However, the Ω parameter is calculated using the average melting temperature ($T_{m}$ = 1724 and 1584 K for the Cr- and Mn-alloys, respectively). When using the peak temperatures measured via DSC, this parameter reduces to Ω* = 0.94 and 0.69. It is important to note that the use of $T_{m}$ implicitly assumes the maximum contribution of the entropic term to the Gibbs energy for the solid solution. Metastable solutions can be obtained through rapid quenching or mechanical alloying processes, but once the system has enough energy (at a given temperature), it will attempt to approach equilibrium. This occurs at much lower temperatures than melting in the samples studied here, and the benefit of $∆S_{conf}$ is not maximized.

When considering the structure of the solid solution, the valence electron concentration, VEC, is typically a key parameter in the various criteria. For the Cr-alloy, VEC = 7.4, whereas for the Mn-alloy, VEC = 7.6. These values are close to the limit from single fcc to multiphase fcc+bcc solid solutions. However, the exact threshold for VEC varies among different authors, ranging from 6.87 [34] to 7.84 [35]. 

Temperature was considered by Wang et al. [26], but regardless of whether this parameter is above or below 90% of $T_{m}$, the VEC values for the studied compositions fall within the bcc+fcc mixed region defined by those authors, below the limit for a single fcc structure (VEC < 7.8).

Application of the criteria proposed by Poletti and Batezzati [36] is not straightforward, as the VEC values are within the limit. Considering the number of conduction electrons per atom (a value of n~2 is obtained for both compositions), a preference for a bcc structure should be expected. However, this is not observed in either the as-milled or annealed samples.

The parameter proposed by Ye et al. [14] is estimated at $T_{m}$ and depends on the packing fraction: $\phi_{Ye}\left(bcc\right)$ < 11 and $\phi_{Ye}\left(fcc\right)$ < 7, values well below the threshold of $\phi_{Ye}$ > 20 for single-phase solid solution. 

To apply the criterion of King et al. [7], $\left|∆G_{max}\right|$ needs to be estimated. Among the different binary alloys of the studied compositions, the minimum enthalpy of formation is obtained for AlNi, with $∆H_{int}$ = −48 kJ/mol [37]. This value is in agreement with the one considered by King et al. [7], although HEAPS software supplies values of −56 and −59 kJ/mol for the Cr- and Mn-alloys, respectively. Following King et al., the entropic contribution is neglected, and $\left|∆G_{max}\right|$~100 kJ/mol, after scaling, whereas $∆G_{mix}=∆H_{mix}-T_{m}∆S_{conf}=$ −34 and −33 kJ/mol for the Cr- and Mn-alloys. This leads to $\phi_{King}$~0.3, clearly below the threshold ($\phi_{King}$>1) that favors the formation of single-phase solid solutions. 

In the frame of our proposed model based on metallic bonding potential, the corresponding values are $U_{0}=-0.25{\langle A\rangle }^{2}/\langle B\rangle =$ −5130 and −4860 kJ/mol; ${∆U}_{0}=$ 10.3 and 20.6 kJ/mol; and $T_{eff}=$ 775 and 1543 K for the Cr- and Mn-alloys, respectively. Both values for $T_{eff}$ are above the threshold of ~500 K for single-phase fcc solid solution. The significantly higher value for the Mn-alloy is in agreement with the higher content of the bcc phase and the observed continuity from a single fcc to mixed fcc+bcc to bcc(B2) microstructures as $T_{eff}$ increases [12]. This model predicts the lattice parameter of the single-phase fcc solid solution to be $a=2\sqrt{2}\langle B\rangle /\langle A\rangle =$ 3.59 and 3.54 Å for the Cr- and Mn-alloys, respectively. These values are lower than the experimental ones but only by ~2%. Using Vegard’s law, which assumes average atomic size, leads to similar values (3.61 and 3.54 Å). A more precise determination of lattice parameters in HEAs has been attempted by other researchers; e.g., Wang et al. [38] reached 0.1% deviation but using an equiatomic HEA composition as the phenomenological value for reference and N-1 parameters (N being the number of elements in the composition) to fit the experimental data.

The criterion for single or multiphase solid solutions based on the $\Lambda =∆S_{conf}/{\delta_{r}}^{2}$ parameter does not consider temperature and takes into account both the benefit of $∆S_{conf}$ and the detrimental effect of elastic energy storage due to the atomic size misfit. In this case, the values of 0.48 and 0.44 Jmol^−1^K^−1^ for the Cr- and Mn-alloys, respectively, place both compositions clearly in the multiphase region (0.24 < Λ < 0.96 Jmol^−1^K^−1^) [6] but far from the development of intermetallics. This parameter can be easily corrected from the excess entropy, as it is proportional to ${\delta_{r}}^{2}$. After corrections, the values for the studied compositions are about 5–10% lower. This correction aligns with the behavior observed in the Mn-alloy after annealing. However, it does not fully account for the development of sigma phase in the Cr-alloy. Tsai et al. proposed a range of 6.88 < VEC < 7.84 [39] for the formation of sigma phase but later added a second criterion based on a minimum content of pair elements able to form sigma phase [40]. Although the former criterion is fulfilled by both studied compositions, only the Cr-alloy presents pairs of elements able to form sigma phase, i.e., Cr-Fe and Cr-Co (Table 1 in [11] collects the different possible pairs). The parameter to account for these paired sigma-forming elements (PSFE) is then 0% for the Mn-alloy (considering the experimental composition, PSFE~4%, but still below the 20% threshold for the possible development of sigma phase [35]) and PSFE = 42% for the Cr-alloy, within the range for certain development of sigma phase (PSFE > 40–45%).

Table 3 provides a comprehensive overview of stability criteria (Ω, Λ, ϕ_Ye_, and $T_{eff}$) for the two studied alloys and a wide range of other alloys produced via mechanical alloying, drawn from various sources in the literature [41,42,43,44,45,46,47,48,49,50,51,52,53,54,55,56,57,58,59,60,61,62,63,64,65,66,67,68,69,70,71,72,73,74,75,76]. The table shows the reported microstructure in the as-milled state as well as in the annealed state. Dispersion of the data can occur due to a shortened milling time (not reaching a stationary situation) or low annealing temperature (not reaching a stable microstructure), but despite this, several points can be discussed. On the one hand, many as-milled samples are out of the predicted microstructure (e.g., $\phi_{Ye}$ < 20 but a single solid solution). However, it is worth noting that these cases generally transition to the predicted microstructure after annealing or spark plasma sintering. On the other hand, in general, those compositions for which the microstructure agrees with the predicted one remain in that stable microstructure after annealing. Exceptions to this are found in the Co_x_CrCuFe(NiMn) series [52,63,66] with $\phi_{Ye}$ > 20. Although these alloys exhibit fcc microstructure in the as-milled state, after annealing, two fcc phases are detected. However, the values of $\phi_{Ye}$ are below 50 and $T_{eff}$ is around the limit value (see Table 3), except for the Mn-free alloy.

Finally, there are some compositions where, despite the predicted composition being a single-phase solid solution, a mixture of phases is observed in the as-milled state. The microstructure obtained by mechanical alloying (i.e., starting from a mixture of pure powders or segregated phases) is strongly dependent on the milling time. In fact, despite a solid solution being possible, for short enough milling times, supersaturated or stable solid solutions are not yet achieved. The main difference for the mechanical alloying route with respect to rapid quenching is that the former develops a supersaturated solution at low temperatures. Therefore, the stability criteria based on high-temperature thermodynamically-stable systems, assuming that they are frozen at low temperatures because of rapid quenching, are not straightforwardly applicable to mechanical alloys. In this sense, most of the stability criteria used for HEAs are based on a maximum effect of configurational entropy on the Gibbs energy (assuming its contribution at the melting temperature). Moreover, an extra complication in mechanical alloyed systems is the possibility of recrystallization effects of the supersaturated solid solutions when the milling time is too long (e.g., due to contamination). 

**Figure 10:**
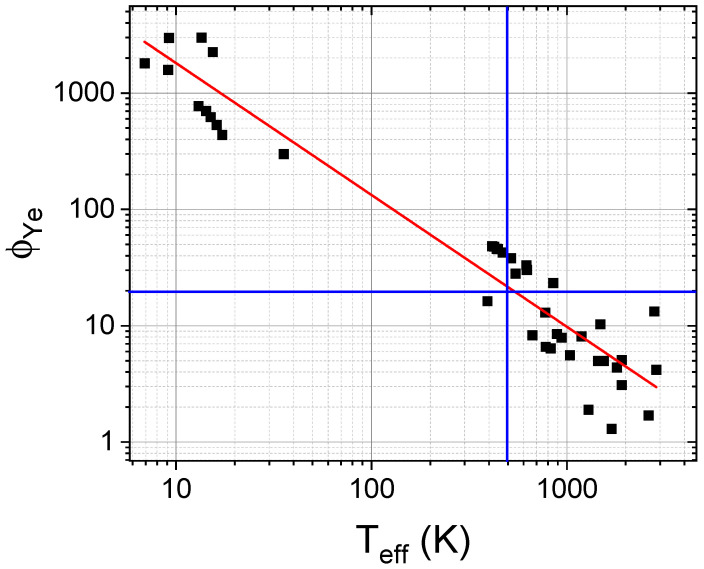
Linear correlation between the logarithms of $\phi_{Ye}$ and $T_{eff}$. Black squares correspond to data taken from Table 3. Red line corresponds to the linear fitting to the data (regression coefficient R=−0.97). Blue lines indicate the limit values for predicting the development of a single-phase solid solution ($\phi_{Ye}$ > 20 and $T_{eff}$ < 500 K).

Table 3 shows that different stability criteria are correlated for a given composition. This is evidenced in Figure 10, where $\phi_{Ye}$ is plotted against $T_{eff}$. The reported regions for single-phase solid solutions, $\phi_{Ye}$ > 20 and $T_{eff}$ < 500 K, are in good agreement.

**Figure 11:**
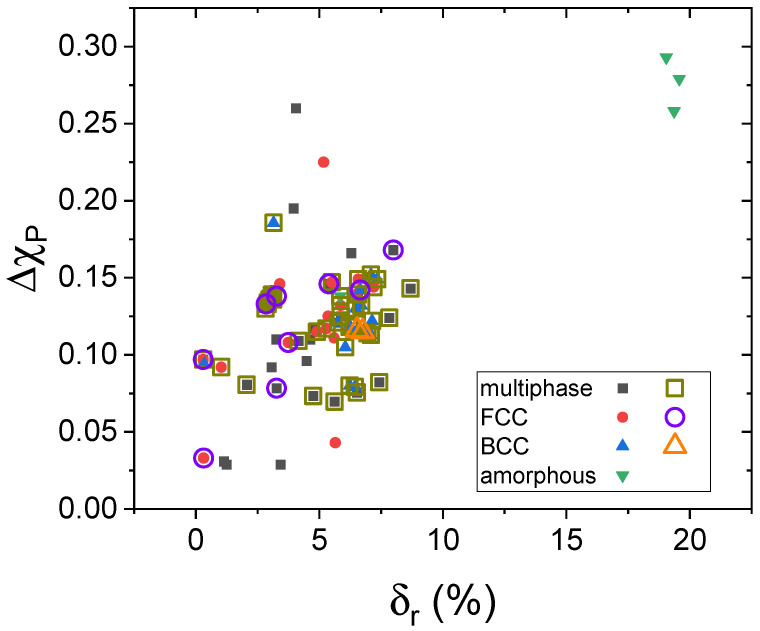
Darken–Gurry plot showing electronegativity difference (using Pauling values) against atomic size misfit, as suggested by Suryanarayana [26]. Solid symbols correspond to as-milled samples and hollow symbols correspond to annealed samples.

Figure 11 shows the Darken–Gurry plot suggested by Suryanarayana [26] as a new criterion for predicting the microstructures of HEAs. However, the plot does not reveal clear demarcation regions between single-phase solid solutions and microstructures formed by mixed phases, neither for the as-milled samples (solid symbols) nor for the annealed ones (hollow symbols enclosing the corresponding solid one of the as-milled counterpart). The absence of distinct regions in the plot emphasizes the complex and multifaceted nature of stability and microstructure formation in HEAs produced via mechanical alloying. This indicates that additional factors or criteria may need to be considered to better understand and predict the behavior of these samples.

## 5. Conclusions

In this work, we showed that high-entropy alloys can be produced via mechanical alloying as single-phase solid solutions even beyond the stability criteria that are currently used to predict the microstructures of multielement alloys.

The microstructure after thermal treatment of supersaturated solutions tends to be closer to the one predicted by applying these stability criteria. Nevertheless, these criteria are generally used assuming a maximum contribution of the configurational entropy to the Gibbs free energy (the entropy contribution is added to the energy balance using the melting temperature). However, the supersaturated solution produced via mechanical alloying destabilizes at temperatures below melting. A good correlation was found for those criteria based on thermodynamics and atomic size misfit with one recently proposed by the current authors and based on the average of the potential coefficients describing the metallic bonding.

In addition to the analysis of several compositions for which data were taken from the literature, in this work, we produced two single-phase metastable fcc solid solutions by means of mechanical alloying with compositions (Al_0.75_CoXFeNi with X = r and Mn) outside of the ranges predicted by the various criteria. Thermal analysis showed that this microstructure is stable up to above 600 K with a better-defined transition in the alloy with Mn. After annealing, both samples developed a bcc phase coexisting with the remaining fcc phase. Moreover, sigma phase was also detected for the Cr-containing alloy. These post-annealing microstructures agree with the predictions from the discussed stability criteria.

In addition to the solid solution stability, magnetic properties were analyzed for these two compositions in the as-milled and annealed stages. The supersaturated fcc solid solutions were paramagnetic at room temperature (except for some impurities probably coming from the milling media and only detected by means of VSM and TMG). After annealing, the biphasic solid solution developed in the Mn-containing alloy exhibited soft magnetism with a high specific saturation magnetization of 100 Am^2^/kg.

## Figures and Tables

**Table 1:** Different criteria proposed in the literature to predict the microstructure of multielement alloys. Data obtained with the help of HEAPS software from Martin et al. [11]. $T_{m}$, melting temperature; $∆S_{conf}$, configurational entropy; $∆H_{mix}$, mixing enthalpy; $S^{xs}$, excess entropy; $∆U_{0}$, excess bonding energy using average coefficients with respect to average over bonding energy of segregated pure metals.

Parameter	Ref	Comment		Range for Single-Phase HEA
$\Omega =\frac{T_{m}∆S_{conf}}{\left|∆H_{mix}\right|}$	[10]	Ratio between entropic and enthalpic contributions to mix		Ω ≥ 1.1 and $\delta_{r}$ ≤ 6.6%
$\Lambda =\frac{∆S_{conf}}{{\delta_{r}}^{2}}$	[6]	Ratio between entropy due to mixing and elastic energy stored due to size misfit		$\Lambda >0.95\;J\;{mol}^{-1}K^{-1}$ and $-5\;kJ\;{mol}^{-1}<∆H_{mix}<0$
$\phi_{Ye}\left(f\right)=\frac{∆S_{conf}-\frac{∆H_{mix}}{T_{m}}}{\left|S^{xs}\left(f\right)\right|}$	[14]	Entropy ratio between mixing and excess energy due to atomic size misfit.	bcc	$\phi_{Ye}>20$
			fcc	
$\phi_{King}=\frac{∆H_{mix}-T_{m}∆S_{conf}}{-\left|∆G_{max}\right|}$	[7]	$\left|∆G_{max}\right|$ is the scaled maximum among absolute values of mixing Gibbs enthalpies of the binary alloys and possible intermetallics		$\phi_{King}>1$
$T_{eff}=\frac{∆U_{0}}{∆S_{conf}}$	[12]	Effective temperature at which entropic term equals bonding energy		$T_{eff}<500\;K$

**Table 2:** Parameters used to describe HEA solid solution stability applied to the two compositions studied here. R, gas constant; $x_{i}$, atomic fraction of element *i*; $∆H_{ij}$, enthalpy of mixing between elements *i* and *j*; $r_{i}$, atomic radius of element *i*; $r=\sum r_{i}/n$, average atomic radius. Data obtained with the help of HEAPS software from Martin et al. [11].

Parameter	Ref	Comment	Units	Cr-Alloy	Mn-Alloy
$∆S_{conf}=-R\sum x_{i}lnx_{i}$	[3]	Configurational entropy. Maximum entropy, assuming complete atomic disorder	J mol^−1^K^−1^	13.3	13.3
$∆H_{mix}=\sum_{i\neq j}4∆H_{ij}x_{i}x_{j}$	[3]	Mixing enthalpy, assuming complete atomic disorder	kJ mol^−1^	−10.9	−12.3
$\delta_{r}=\sqrt{\sum_{i=1}^{n}x_{i}{\left(1-\frac{r_{i}}{r}\right)}^{2}}$	[6]	Atomic size misfit: ${\delta_{r}}^{2}$ should be related to the lattice strain energy	%	5.29	5.51
$S^{xs}\left(bcc\right)=-0.022992(6)\cdot {\delta_{r}}^{2}$	[16] (*)	Correction to $∆S_{conf}$; $S^{xs}<0$ due to preferential location of smaller atoms close to larger ones. Linearity estimated for CoFeNiMn(x)Cr(y)Al(z)	J mol^−1^K^−1^	−0.65	−0.68
$S^{xs}\left(fcc\right)=-0.03720\left(1\right)\cdot {\delta_{r}}^{2}$			J mol^−1^K^−1^	−1.06	−1.11
$\delta_{\chi }=\sqrt{\sum_{i=1}^{n}x_{i}{\left(1-\frac{\chi_{i}}{\chi }\right)}^{2}}$	[28]	Allen electronegativity misfit	%	5.89	4.91
		Pauling		6.56	8.34
$∆VEC=\sqrt{\sum_{i=1}^{n}x_{i}{\left(VEC-{VEC}_{i}\right)}^{2}}$	[33]	VEC is the valence electron concentration number of electrons per atom in incomplete shells		2.35	2.25
$\Omega =\frac{T_{m}∆S_{conf}}{\left|∆H_{mix}\right|}$	[10]	Ratio between entropic and enthalpic contributions to the solid solution		2.11	1.72
$\Lambda =\frac{∆S_{conf}}{{\delta_{r}}^{2}}$	[6]	Ratio between entropy due to mixing and elastic energy stored due to atomic size misfit	J mol^−1^K^−1^	0.476	0.439
$\phi_{Ye}\left(f\right)=\frac{∆S_{conf}-\frac{∆H_{mix}}{T_{m}}}{\left|S^{xs}\left(f\right)\right|}$	[14]	Ratio between entropic term favoring solid solution and excess energy due to atomic size misfit.		6.63	5.02
$\phi_{King}=\frac{∆H_{mix}-T_{m}∆S_{conf}}{-\left|∆G_{max}\right|}$	[7]	$\left|∆G_{max}\right|$ corresponds to the scaled maximum absolute value among the mixing Gibbs enthalpies of the different binary alloys and the possible binary intermetallics		~0.3	~0.3
$T_{eff}=\frac{∆U_{0}}{∆S_{conf}}$	[12]	Effective temperature at which entropic term equals bonding energy	K	775	1543

* Expressions correspond to linear fittings of Figure 1. The correct and more complex equation for $S^{xs}$ is reported in [16].

**Table 3:** Values of the different parameters used to predict multicomponent microstructures for different compositions from the literature.

Composition	As-Milled	Ω	Λ	$\phi_{Ye}$	$T_{eff}$/K	Annealed	Ref.
FeCoNiMnAl_0.75_	fcc	1.7	0.44	5.0	1544	fcc+bcc	This work
FeCoNiCrAl_0.75_	fcc	2.1	0.48	6.6	777	fcc+bcc+σ	
ZrFeNiSi_0.4_B_0.6_	amorphous+fcc	0.5	0.03			*not reported*	[41]
Zr_1.5_FeNiSi0._4_B_0.6_	amorphous+fcc	0.4	0.03			*not reported*	[41]
Zr_2.5_FeNiSi_0.4_B_0.6_	amorphous+fcc	0.4	0.03			*not reported*	[41]
Al_35_Cr_14_Mg_6_Ti_35_V_10_	bcc	1.1	0.30	1.3	1692	bcc(1)+bcc(2)+hcp	[42]
Fe_30_Ni_30_Al_15_Cr_20_Mn_5_	fcc	2.0	0.43	5.6	1035	*not reported*	[43]
CoCrFeNi	fcc	5.8	130	2988	9.2	*not reported*	[44]
AlCoCrFeNi	fcc+bcc	1.8	0.40		937	B2+fcc	[45]
CoCrFeMnNi	fcc	5.8	1.25	28.1	546	fcc	[46]
Al_5_Cu_5_Ni_30_Cr_30_Fe_30_	bcc+fcc	4.7	1.08		332	*not reported*	[47]
Al_12.5_Cu_12.5_Ni_25_Cr_25_Fe_25_	fcc+bcc	5.2	0.56		663	*not reported*	[47]
AlCoCrFeNi	bcc	1.8	0.40	7.9	937	B2+L1_2_+σ	[48]
AlCuFeMnTiV	bcc/amorphous	2.3	0.44		2336	bcc+B2+fcc+hcp	[49]
Al_0.5_CoCrCuFeNi	fcc(1)+fcc(2)+bcc	16.4	0.85		431	fcc(1)+fcc(2)+bcc *	[50]
Al_1.5_CoCrCuFeNi	fcc(1)+fcc(2)+bcc	3.3	0.43		902	fcc(1)+fcc(2)+bcc *	[50]
Al_2.5_CoCrCuFeNi	B2	2.2	0.34	8.1	1185	B2+fcc *	[50]
Al_4_CoCrCuFeNi	B2	1.6	0.29	5.0	1442	B2 *	[50]
Al_20_Li_20_Mg_10_Sc_20_Ti_30_	fcc	42.6	0.48	13.0	773	hcp	[51]
CoCrCuFeMn	fcc	5.6	1.35	30.3	623	fcc(1)+fcc(2)+σ	[52]
CoCrCuFeMnNi_0.5_	fcc	9.9	1.56	38.2	517	fcc(1)+fcc(2)+σ	[52]
CoCrCuFeMnNi	fcc	17.8	1.66	42.6	469	fcc(1)+fcc(2)	[52]
CoCrCuFeMnNi_1.5_	fcc	41.5	1.73	45.8	437	fcc(1)+fcc(2)	[52]
CoCrCuFeMnNi_2_	fcc	100	1.79	48.3	414	fcc	[52]
FeCoNiAl	bcc	1.3	0.30	1.9	1288	bcc+fcc	[53]
FeCoNiAlSi_0.2_	bcc	1.1	0.30	0.5	--	bcc	[53]
FeCoNiAlSi_0.4_	bcc	0.9	0.28	−0.7	--	bcc	[53]
FeCoNiAlSi_0.6_	bcc	0.8	0.27	−1.6	--	bcc+B2	[53]
FeCoNiAlSi_0.8_	bcc	0.8	0.27	−2.3	--	bcc+B2	[53]
Ni_35_Co_35_Cr_12.6_Al_7.5_Ti_5_Mo_1.7_W_1.4_Nb_1.0_Ta_0.5_	fcc+bcc	1.8	0.44		1214	fcc	[54]
FeNiMnCu	fcc	6.7	1.00	23.3	850	*not reported*	[55]
FeNiMnCuCo	fcc	12.4	1.33	33.2	620	*not reported*	[55]
FeNiMnCuCr	fcc+bcc	8.5	1.31		599	*not reported*	[55]
FeNiMnCuMo	bcc+fcc	6.0	0.85		1687	*not reported*	[55]
FeNiMnCuTi	fcc+(bcc)	2.4	0.34		2803	*not reported*	[55]
FeNiMnCuW	bcc+(fcc)	4.7	0.82		2006	*not reported*	[55]
Co_0.18_Cr_0.20_Fe_0.24_Ni_0.19_Ti_0.19_	bcc+fcc	1.7	0.31		1853	fcc(1)+fcc(2) *	[56]
CoCrFeNi	fcc	5.8	130	2988	9.2	fcc+Cr_7_C_3_	[57]
Al_0.3_CoCrFeNi	fcc	3.2	0.91	16.3	392	fcc+Cr_7_C_3_	[57]
Al_0.6_CoCrFeNi	fcc	2.4	0.55	8.3	665	fcc+bcc+Cr_7_C_3_	[57]
AlCoCrFeNi	bcc+fcc	1.8	0.40		937	fcc+bcc+Cr_7_C_3_	[57]
AlCuSiFeCr	bcc	1.2	0.26	2.2	--	bcc +fcc+σ	[58]
AlCuSiFeMn	bcc	0.9	0.25	−0.9	--	bcc +fcc+μ	[58]
AlCuSiFeZn	bcc+fcc	1.3	0.22		--	fcc+bcc	[58]
AlCuSiFeSn	bcc+fcc	1.8	0.18		--	Fcc *	[58]
NiCoCrFe	bcc+fcc	5.8	130		9.2	fcc+bcc	[59]
NiCoCrFeZr0._4_	bcc+fcc	1.8	0.20		2803	fcc	[59]
CuNiCoZnAl	fcc	2.5	0.39	6.4	823	multiphase	[60]
Fe_40_Mn_14_Ni_10_Cr_10_Al_15_C_1_	bcc	1.9	0.25	5.5	--	fcc+B2+Cr_3_Si *	[61]
CrNbTiVZn	fcc	7.4	0.42	10.3	1480	*not reported*	[62]
CoCrCuFeNi	fcc	7.4	12.6	300	35.5	fcc(1)+fcc(2)	[63]
TiFeNiCr	fcc	1.4	0.22	1.7	2615	fcc(1)+fcc(2)+σ	[64]
TiFeNiCrMn	fcc	2.0	0.31	4.2	2868	fcc(1)+fcc(2)+σ	[64]
TiFeNiCrCo	fcc	1.6	0.30	3.1	1907	fcc	[64]
CrMnFeVTi	bcc	5.6	0.37	13.3	2796	bcc+fcc	[65]
Co_0.5_CrCuFeMnNi	fcc+bcc	12.8	1.54		508	fcc(1)+fcc(2) *	[66]
CoCrCuFeMnNi	fcc	17.8	1.66	42.6	469	fcc(1)+fcc(2)	[66]
Co_1.5_CrCuFeMnNi	fcc	24.6	1.75	45.6	443	fcc(1)+fcc(2)	[66]
Co_2_CrCuFeMnNi	fcc	34.3	1.82	47.9	424	fcc(1)+fcc(2)	[66]
CoFeNi	fcc	12.1	88.6	2257	15.4	fcc+Cr_7_C_3_	[67]
CoCrFeNi	fcc	5.8	130	2988	9.2	fcc+Cr_7_C_3_	[67]
CoCrFeMnNi	fcc	5.8	1.25	28.1	546	fcc	[67]
CoCrFe	bcc	6.6	78.9	3008	13.5	*not reported*	[68]
CoCrFeNi	fcc	5.8	130	2988	9.2	*not reported*	[68]
CoCrFeNiTi	bcc	1.6	0.30	5.1	1907	*not reported*	[68]
CoCrFeNiZn	fcc+bcc	6.8	0.62		161	*not reported*	[68]
CoCrFeNiSi	fcc+bcc	0.9	1.42		--	*not reported*	[68]
CoCrFeNiAl	bcc	1.8	0.40	7.9	937	*not reported*	[68]
CoCuFe	fcc+bcc	1.9	5.96		65	*not reported*	[68]
CoCuFeNi	fcc(1)+fcc(2)	3.8	8.90		49	*not reported*	[68]
CoCuFeNiTi	fcc	2.1	0.32	4.4	1799	*not reported*	[68]
CoCuFeNiZn	fcc(1)+fcc(2)	11.2	0.66		164	*not reported*	[68]
CoCuFeNiSi	fcc(1)+fcc(2)	1.3	1.13		--	*not reported*	[68]
CoCuFeNiAl	fcc	3.9	0.43	8.5	889	*not reported*	[68]
AlLiMg_0.5_Ti_1.5_	hcp+AlLi	4.0	0.84	17.2	437	intermetallics	[69]
(AlLiMg_0.5_Ti_1.5_)_95_Sc_5_	hcp	6.7	0.69	16.1	532	intermetallics	[69]
(AlLiMg_0.5_Ti_1.5_)_90_Sc_10_	hcp	12.7	0.59	15.0	623	intermetallics	[69]
(AlLiMg_0.5_Ti_1.5_)_85_Sc_15_	hcp	44.4	0.53	14.2	703	intermetallics	[69]
(AlLiMg_0.5_Ti_1.5_)_80_Sc_20_	hcp	42.6	0.48	13.0	773	hcp+intermet.	[69]
Al_0.5_CrFeNiTi	fcc+bcc+hcp	1.2	0.24		2279	fcc+bcc+2 hcp	[70]
FeCoCrNiMn	fcc+bcc	5.8	1.25		546	fcc	[71]
(FeCoCrNi)_80_Mn_10_Al_10_	fcc+bcc	2.8	0.65		800	fcc+bcc+σ	[71]
CoCrMnNbTi	bcc(1)+bcc(2)+tet	2.2	0.32			*not reported*	[72]
CoCrFeNiW_0.2_	bcc+fcc	7.0	2.95		433	bcc+fcc+σ	[73]
CrFeNiTiV	bcc	2.0	0.32	6.9	1802	bcc+Ni_3_Ti *	[74]
AlCrFeNiCu	bcc+fcc	5.4	0.42		912	B2+fcc+σ	[75]
AlCrFeNbMo	bcc	2.3	0.35	9.1	1587	bcc+fcc+σ	[75]
WNbMoVTa	bcc+WC	8.5	1.35			bcc+others	[76]
WNbMoVTaCr	bcc+WC+NbTa	9.0	0.69			bcc+others	[76]
WNbMoVTaAl	bcc+WC+NbTa	4.0	1.49			bcc+others	[76]
WNbMoVTaCr_0.5_	bcc+WC+NbTa	9.1	0.87			bcc+others	[76]
WNbMoVTaAl_0.5_	bcc+WC+NbTa	5.2	1.46			bcc+others	[76]
WNbMoVTaCr_0.5_Al_0.5_	bcc+WC+NbTa	5.7	0.94			bcc+others	[76]
WNbMoVTaCr_0.5_Al	bcc+WC+NbTa	4.4	0.96			bcc+others	[76]
WNbMoVTaCrAl_0.5_	bcc+WC+NbTa	5.9	0.73			bcc+others	[76]
WNbMoVTaCrAl	bcc+WC+NbTa	4.5	0.75			bcc+others	[76]

* There are other phases detected.

## Data Availability

Data are available on request to the authors.
